# Capacitation and acrosome reaction differences of bovine, mouse and porcine spermatozoa in responsiveness to estrogenic compounds

**DOI:** 10.1186/2055-0391-56-26

**Published:** 2014-11-27

**Authors:** Do-Yeal Ryu, Ye-Ji Kim, June-Sub Lee, Md Saidur Rahman, Woo-Sung Kwon, Sung-Jae Yoon, Myung-Geol Pang

**Affiliations:** Department of Animal Science and Technology, Chung-Ang University, 4726 Seodong-daero, Anseong, 456-756 Gyeonggi-Do Republic of Korea

**Keywords:** Capacitation, Acrosome reaction, Estrogen, Endocrine disruptor, Spermatozoa

## Abstract

**Background:**

Endocrine disruptors are exogenous substance, interfere with the endocrine system, and disrupt hormonal functions. However, the effect of endocrine disruptors in different species has not yet been elucidated. Therefore, we investigated the possible effects of 17ß-estradiol (E2), progesterone (P4), genistein (GEN) and 4-tert-octylphenol (OP), on capacitation and the acrosome reaction in bovine, mouse, and porcine spermatozoa. In this *in vitro* trial, spermatozoa were incubated with 0.001-100 μM of each chemical either 15 or 30 min and then assessed capacitation status using chlortetracycline staining.

**Results:**

E2 significantly increased capacitation and the acrosome reaction after 30 min, while the acrosome reaction after 15 min incubation in mouse spermatozoa. Simultaneously, capacitation and the acrosome reaction were induced after 15 and 30 min incubation in porcine spermatozoa, respectively. Capacitation was increased in porcine spermatozoa after 15 min incubation at the lowest concentration, while the acrosome reaction was increased in mouse spermatozoa after 30 min (*P* <0.05). E2 significantly increased the acrosome reaction in porcine spermatozoa, but only at the highest concentration examined (*P* <0.05). P4 significantly increased the acrosome reaction in bovine and mouse spermatozoa treated for 15 min (*P* <0.05). The same treatment significantly increased capacitation in porcine spermatozoa (*P* <0.05). P4 significantly increased capacitation in mouse spermatozoa treated for 30 min (*P* <0.05). GEN significantly increased the acrosome reaction in porcine spermatozoa treated for 15 and 30 min and in mouse spermatozoa treated for 30 min (*P* <0.05). OP significantly increased the acrosome reaction in mouse spermatozoa after 15 min (*P* <0.05). Besides, when spermatozoa were incubated for 30 min, capacitation and the acrosome reaction were higher than 15 min incubation in E2 or GEN. Furthermore, the responsiveness of bovine, mouse and porcine spermatozoa to each chemical differed.

**Conclusions:**

In conclusion, all chemicals studied effectively increased capacitation and the acrosome reaction in bovine, mouse, and porcine spermatozoa. Also we found that both E2 and P4 were more potent than environmental estrogens in altering sperm function. Porcine and mouse spermatozoa were more responsive than bovine spermatozoa.

## Background

Estrogens play key roles in regulating various physiological phenomena related to normal growth, development, and reproduction in mammals [1]. Although estrogens have been considered to be female reproductive hormones, recent evidence has indicated that they play an important role in the development and regulation of the male reproductive system [2]. Estrogens were detected in male serum as well as in the male gonad [3].

Recent reports have shown that endocrine disruptors are exogenous substances that interfere with the endocrine system and might disrupt hormone function in various wildlife species. Moreover, it has also been suggested that these compounds may be responsible for a variety of reproductive disturbances in men, including possible declines in sperm concentration [4, 5]. Many non-steroid compounds in the environment have been found to exhibit estrogenic activity [6, 7], where these include natural phytoestrogens, pesticides, and industrial products with various homologies to estradiol that can bind with estrogen receptors, acting either as agonists or antagonists [8]. Most studies of xenobiotics have focused on long-term developmental effects on the testis, the male reproductive tract and semen quality. When xenobiotics are used at relatively high dosages, they disrupt spermatogenesis and hence decrease overall male fertility [9].

Luconi et al. [2] have identified and characterized novel nongenomic estrogen receptors on the cell membrane of human spermatozoa that interfere with the effects of progesterone (P4). Thus, spermatozoa may represent a suitable model to study the possible effects of estrogenic xenobiotics on the function of spermatozoa. Adeoya-Osiguwa et al. [10] recently reported evidence that very low dosages of several xenobiotics have direct effects on the function of spermatozoa, significantly accelerating capacitation and the acrosome reaction in mice. However, there are no data on these effects in domestic animals. Examination of the physiological and nonphysiological effects of estrogenic xenobiotics on sperm function requires a high-quality *in vitro* test system [11].

To look for any significant effects of treatment length and concentration on the rate of capacitation and acrosome reaction, we examined the effects of 17β-estradiol (E2), P4, and two estrogenic compounds, namely genistein (GEN) and 4-tert-octylphenol (OP) on bovine, mouse and porcine spermatozoa.

## Methods

All procedures were performed according to guidelines for the ethical treatment of animals and approved by Institutional Animal Care and Use Committee of Chung-Ang University (Approval no. 12-0021).

### Medium and reagents

Throughout this study, bovine, mouse and porcine spermatozoa were treated in modified TCM 199 that comprised TCM 199 with Earle’s salts containing 10% heat-inactivated fetal calf serum (v/v), 0.91 mM sodium pyruvate, 3.05 mM D-glucose, 2.92 mM calcium lactate, 50 IU/l penicillin G, and 30 μg/ml streptomycin sulfate. A stock solution of 1000 μM E2 and P4 (Sigma-Aldrich, St Louis, MO, USA) was prepared in dimethyl sulfoxide (DMSO) and stored at −20°C. Working stock solutions were prepared daily by first diluting the initial stock solution in 10% DMSO: 0.9% NaCl (1:1). This solution was used for subsequent dilutions of the standard medium. The other stock solutions (100 μM) of GEN (Sigma-Aldrich, St Louis, MO, USA), P4 and OP (Sigma-Aldrich, St Louis, MO, USA) were prepared in absolute ethanol and stored at −20°C. The working stock solutions were prepared daily using standard medium as diluent.

### Preparation of spermatozoa

Frozen bovine semen and liquid porcine semen were obtained from the National Agriculture Cooperation Federation (Goyang, Gyeonggi, Korea) and Yonam Genetics, Inc. (Chonan, Chungnam, Korea), respectively. Epididymal mouse sperm cells were collected from 9-week-old male ICR mice (Central Lab. Animal Inc, Seoul, Korea). For frozen-thawed bovine and liquid porcine semen, sperm cells were centrifuged at 500 × g for 3 min and the sperm pellets were diluted with modified TCM 199 with or without chemicals. Subsequently, suspensions were centrifuged at 500 × g for 3 min and sperm pellets were diluted with modified TCM 199 solution containing one of the chemicals examined. These were incubated for 15 min or 30 min in an atmosphere of 5% CO_2_ at 39°C. For mouse spermatozoa, caudal epididymal spermatozoa from three mature ICR males were released into sterile plastic dishes containing modified TCM 199 (0.1% BSA). Suspensions were then allowed to disperse for 5 min on a warming tray and motile sperm cells were collected. Sperm cells were diluted with modified TCM 199 containing one of the chemicals examined. These samples were incubated for 15 min or 30 min in an atmosphere of 5% CO_2_ at 37°C. The analysis of each condition for each animal was replicated 3 times.

### Combined Hoechst 33258/chlortetracycline fluorescence assessment of spermatozoa (H33258/CTC)

The dual staining method performed was based on that described by Perez et al. [12], with some modifications. Briefly, 135 μl of semen (2 × 10^8^ cell/ml) was added to 15 μl of H33258 solution (10 μg H33258/ml D-PBS) and incubated at 37°C for 10 min in a light-shielded water bath. Excess dye was removed by layering the mixture over 250 μl of 2% (w/v) polyvinylpyrrolidone (PVP) in PBS that had been centrifuged at 400 × g for 10 min. The supernatant was discarded and the pellet was resuspended in 700 μl of PBS and 500 μl of this solution was added to 500 μl of a freshly prepared CTC solution (1.3 mg CTC in 5 ml buffer: 20 μM Tris, 130 μM NaCl, 5 μM cystein). After 20 sec, the reaction was stopped by the addition of 10 μl of 12.5% (v/v) glutaraldehyde solution in 1 M Tris buffer and maintained at 4°C in the dark until evaluation (within 24 h of preparation). Samples were observed with a Nikon microphot-FXA under epifluorescence illumination using UV BP 340-380/LP 425 and BP 450-490/LP 515 excitation/emission filters for H33258 and CTC, respectively. Spermatozoa were classified as shown in Table 1: dead (D type, when nuclei showed bright blue fluorescence over the sperm head), live noncapacitated (F type, bright green fluorescence distributed uniformly over the entire sperm head, with or without stronger fluorescent line at the equatorial segment), live capacitated (B type, green fluorescence over acrosomal region and dark postacrosome), or live acrosome-reacted (AR type, spermatozoa showing a mottled green fluorescence over head, green fluorescence only in post acrosomal region or no fluorescence on the head) [13]. All spermatozoa had bright green fluorescent midpieces. Two slides per sample were evaluated, with at least 100 spermatozoa per slide.

**Table 1:** **Determination of capacitation status patterns**

Patterns (%)	Description
F	Full fluorescence characteristic of ejaculated spermatozoa: characteristic of uncapacitated spermatozoa
B	Banded, indicative of capacitated spermatozoa and fluorescence only in the post acrosomal region: characteristic of capacitated spermatozoa
AR	Spermatozoa showing a mottled green fluorescence over head, green fluorescence only in post acrosomal region or no fluorescence on the head: typical acrosome-reacted spermatozoa

### Statistics

The data were analyzed using ANOVA performed with SPSS software (v. 12.0; Chicago, Illinois, USA). This test compares responses within replicates for a significant difference to be obtained, a consistent and reasonable magnitude is required between control and treated samples. A value of *P* <0.05 was considered statistically significant.

## Results

This study investigated the possible effects of E2, P4, and two estrogenic environmental estrogens, GEN, and OP, on capacitation and acrosome reaction in bovine, mouse, and porcine spermatozoa *in vitro*. Bovine and porcine spermatozoa suspensions were incubated with 0.001-100 μM E2, P4, GEN, and OP for either 15 or 30 min at 39°C and then assessed using CTC fluorescence. Mouse spermatozoa were incubated at 37°C.

### Effects of E2 on bovine, mouse, and porcine spermatozoa

**Figure 1:**
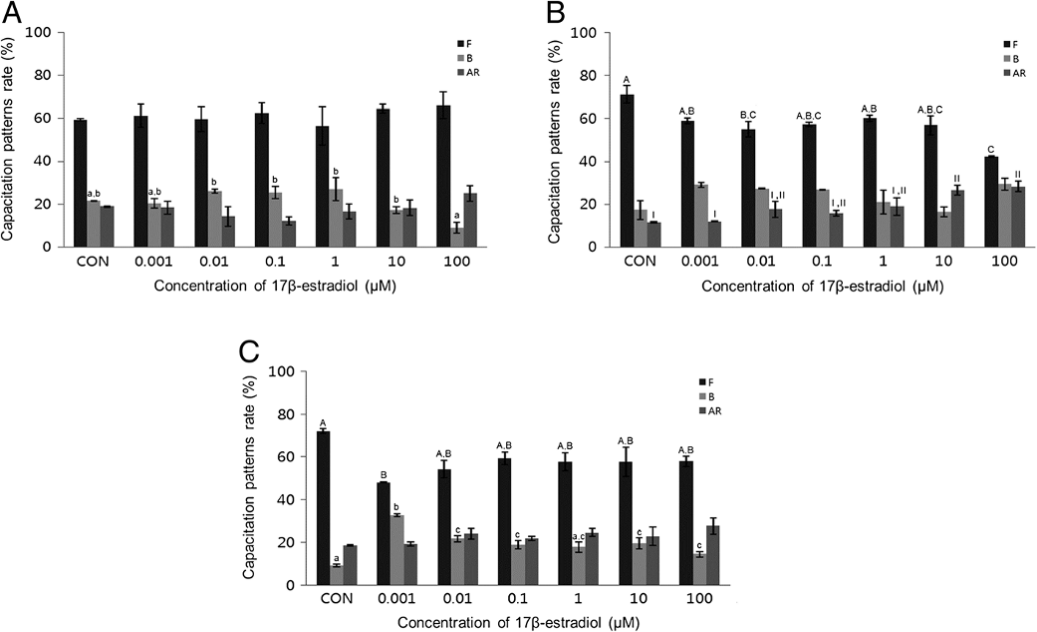
**Effects of 15 min of incubation with 17β-estradiol (E2) on capacitation status. (A)** Change of sperm capacitation status of bovine spermatozoa in the absence or presence of E2 (0.001 to 100 μM). **(B)** Change of sperm capacitation status of mouse spermatozoa in the absence or presence of E2 (0.001 to 100 μM). **(C)** Change of sperm capacitation status of porcine spermatozoa under in the absence or presence of E2 (0.001 to 100 μM). Capacitation status was distinguished F, B and AR pattern (Black Bar: F pattern, Grey Bar: B pattern, Dark-grey Bar: AR pattern). Data represent mean ± SEM, n =3. ^A, B, C^ Values with different superscripts were significantly different compared to control and the F pattern group, by ANOVA (*P* <0.05). ^a, b, c^ Values with different superscripts were significantly different compared to control and the B pattern group, by ANOVA (*P* <0.05). ^I, II^ Values with different superscripts were significantly different compared to control and the AR pattern group, by ANOVA (*P* <0.05).

**Figure 2:**
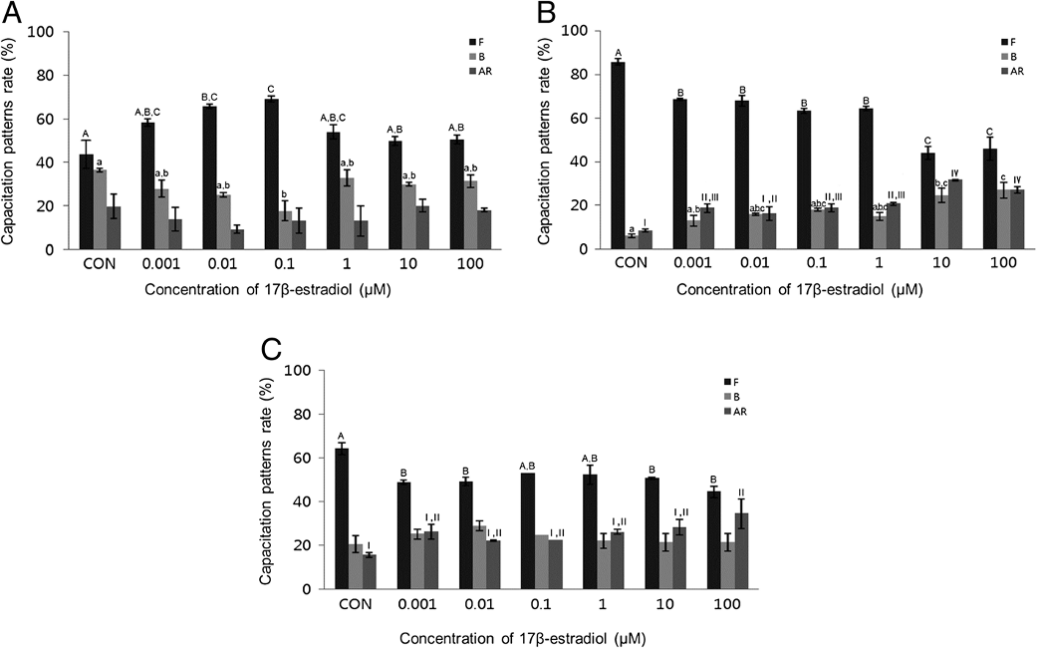
**Effects of 30 min of incubation with 17β-estradiol (E2) on capacitation status. (A)** Change of sperm capacitation status of bovine spermatozoa in the absence or presence of E2 (0.001 to 100 μM). **(B)** Change of sperm capacitation status of mouse spermatozoa in the absence or presence of E2 (0.001 to 100 μM). **(C)** Change of sperm capacitation status of porcine spermatozoa in the absence or presence of E2 (0.001 to 100 μM). Capacitation status was distinguished F, B and AR pattern (Black Bar: F pattern, Grey Bar: B pattern, Dark-grey Bar: AR pattern). Data represent mean ± SEM, n =3. ^A, B, C^ Values with different superscripts were significantly different compared to control and the F pattern group, by ANOVA (*P* <0.05). ^a, b, c^ Values with different superscripts were significantly different compared to control and the B pattern group, by ANOVA (*P* <0.05). ^I, II, III, IV^ Values with different superscripts were significantly different compared to control and the AR pattern group, by ANOVA (*P* <0.05).

A concentration-dependent pattern of capacitation was observed in mouse spermatozoa. E2 (0.001-100 μM) significantly increased the acrosome reaction in mouse spermatozoa and capacitation in porcine spermatozoa after 15 min incubation (*P* <0.05, Figure 1B and C). Following 30 min exposure, E2 significantly increased both capacitation and the acrosome reaction in mouse spermatozoa, and significantly increased the acrosome reaction in porcine spermatozoa (*P* <0.05) (Figure 2B and C). No detectable effects were observed in bovine spermatozoa treated for 15 min or 30 min (Figures 1A and 2A). The acrosome reaction in mouse spermatozoa was responded to E2 at higher concentrations (10–100 μM) after 15 min incubation and responded to 0.001 μM after 30 min incubation. Capacitation in mouse spermatozoa was increased significantly at higher concentrations (10–100 μM) after 30 min incubation (*P* <0.05) (Figures 1B and 2B). Capacitation was increased in porcine spermatozoa after 15 min incubation in E2 at a concentration of 0.001 μM. This effect decreased gradually with increasing doses (Figure 1C). E2 significantly increased the acrosome reaction in porcine spermatozoa, but only after 30 min incubation at the highest concentration (*P* <0.05) (Figures 1C and 2C).

### Effects of P4 on bovine, mouse, and porcine spermatozoa

**Figure 3:**
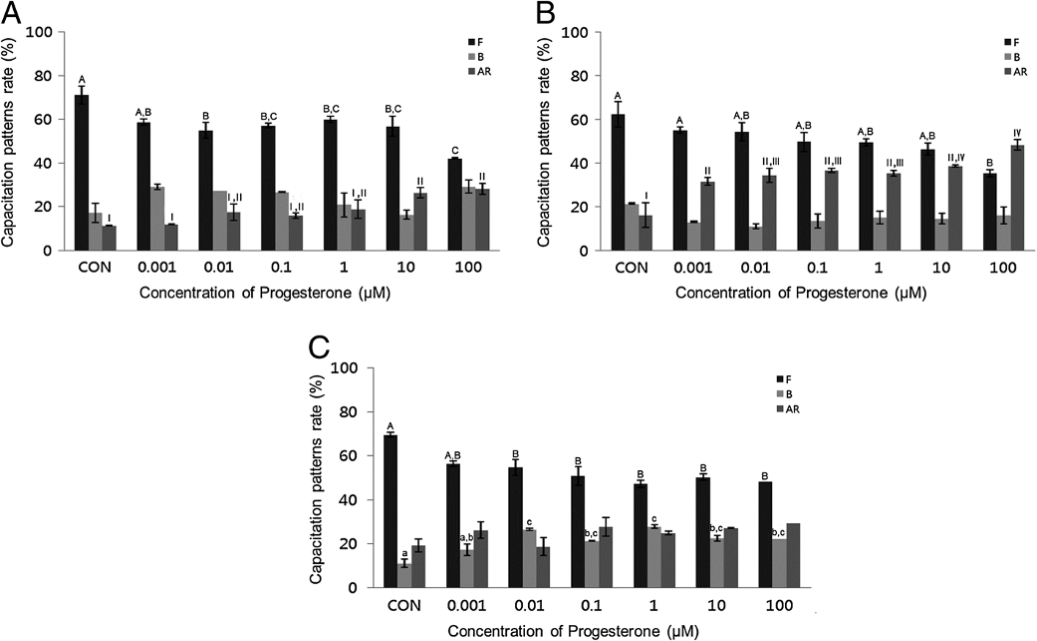
**Effects of 15 min of incubation with progesterone (P4) on capacitation status. (A)** Change of sperm capacitation status of bovine spermatozoa in the absence or presence of P4 (0.001 to 100 μM). **(B)** Change of sperm capacitation status of mouse spermatozoa in the absence or presence of P4 (0.001 to 100 μM). **(C)** Change of sperm capacitation status of porcine spermatozoa in the absence or presence of P4 (0.001 to 100 μM). Capacitation status was distinguished F, B and AR pattern (Black Bar: F pattern, Grey Bar: B pattern, Dark-grey Bar: AR pattern). Data represent mean ± SEM, n =3. ^A, B, C^ Values with different superscripts were significantly different compared to control and the F pattern group, by ANOVA (*P* <0.05). ^a, b, c^ Values with different superscripts were significantly different compared to control and the B pattern group, by ANOVA (*P* <0.05). ^I, II, III, IV^ Values with different superscripts were significantly different compared to control and the AR pattern group, by ANOVA (*P* <0.05).

**Figure 4:**
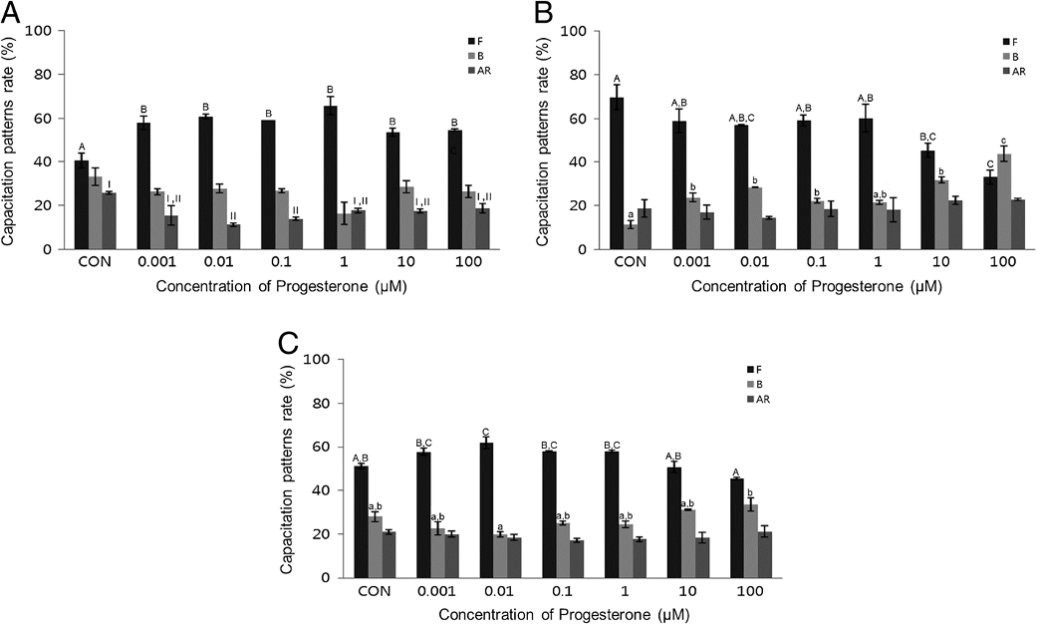
**Effects of 30 min of incubation with progesterone (P4) on capacitation status. (A)** Change of sperm capacitation status of bovine spermatozoa in the absence or presence of P4 (0.001 to 100 μM). **(B)** Change of sperm capacitation status of mouse spermatozoa in the absence or presence of P4 (0.001 to 100 μM). **(C)** Change of sperm capacitation status of porcine spermatozoa in the absence or presence of P4 (0.001 to 100 μM). Capacitation status was distinguished F, B and AR pattern (Black Bar: F pattern, Grey Bar: B pattern, Dark-grey Bar: AR pattern). Data represent mean ± SEM, n =3. ^A, B, C^ Values with different superscripts were significantly different compared to control and the F pattern group, by ANOVA (*P* <0.05). ^a, b, c^ Values with different superscripts were significantly different compared to control and the B pattern group, by ANOVA (*P* <0.05). ^I, II^ Values with different superscripts were significantly different compared to control and the AR pattern group, by ANOVA (*P* <0.05).

After a 15-minute exposure to P4, a concentration-dependent effect on acrosome reaction was observed. P4 (0.001-100 μM) significantly increased the acrosome reaction in both bovine and mouse spermatozoa treated for 15 min (*P* <0.05). This condition also significantly increased capacitation (*P* <0.05) (Figure 3A and B). No detectable acrosome reaction was observed in porcine spermatozoa (Figure 3C). P4 significantly increased capacitation in a dose-dependent manner in mouse spermatozoa treated for 30 min (*P* <0.05) (Figure 4B), while no detectable effects were observed in bovine or porcine spermatozoa (Figure 4A and C).

In bovine spermatozoa, the acrosome reaction was significantly increased after 15 min incubation at higher concentrations (*P* <0.05) (Figure 3A) (10–100 μM). The mouse spermatozoa first exhibited a response at 0.001 μM (Figure 3B). However, no significant effects on acrosome reaction were observed after 30 min incubation in any treated spermatozoa (Figure 4).

### Effects of GEN on bovine, mouse, and porcine spermatozoa

**Figure 5:**
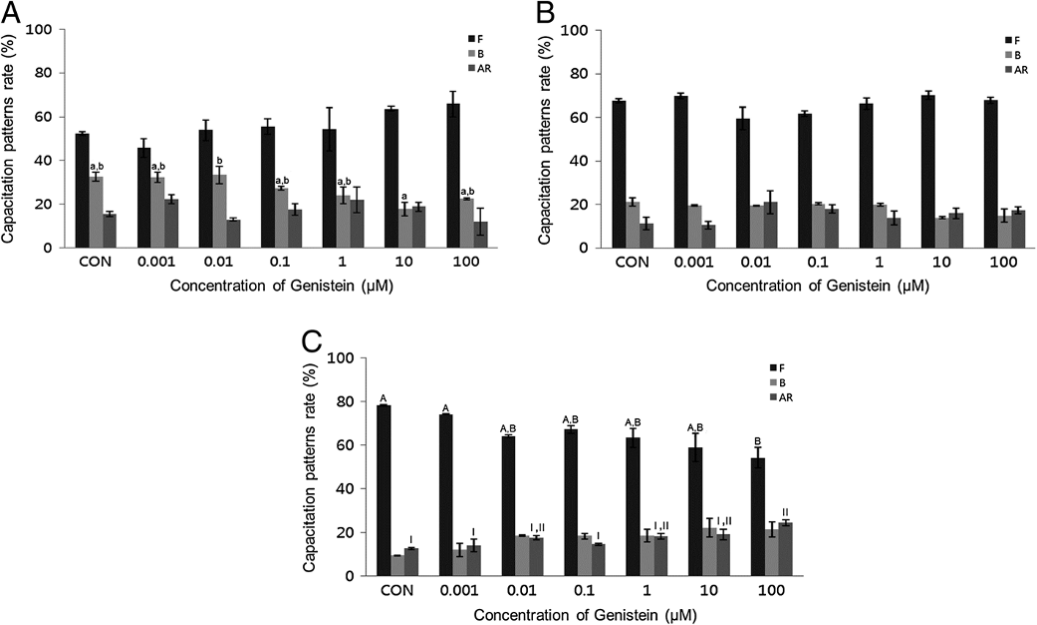
**Effects of 15 min of incubation with genistein (GEN) on capacitation status. (A)** Change of sperm capacitation status of bovine spermatozoa in the absence or presence of GEN (0.001 to 100 μM). **(B)** Change of sperm capacitation status of mouse spermatozoa in the absence or presence of GEN (0.001 to 100 μM). **(C)** Change of sperm capacitation status of porcine spermatozoa in the absence or presence of GEN (0.001 to 100 μM). Capacitation status was distinguished F, B and AR pattern (Black Bar: F pattern, Grey Bar: B pattern, Dark-grey Bar: AR pattern). Data represent mean ± SEM, n =3. ^A, B^ Values with different superscripts were significantly different compared to control and the F pattern group, by ANOVA (*P* <0.05). ^a, b^ Values with different superscripts were significantly different compared to control and the B pattern group, by ANOVA (*P* <0.05). ^I, II^ Values with different superscripts were significantly different compared to control and the AR pattern group, by ANOVA (*P* <0.05).

**Figure 6:**
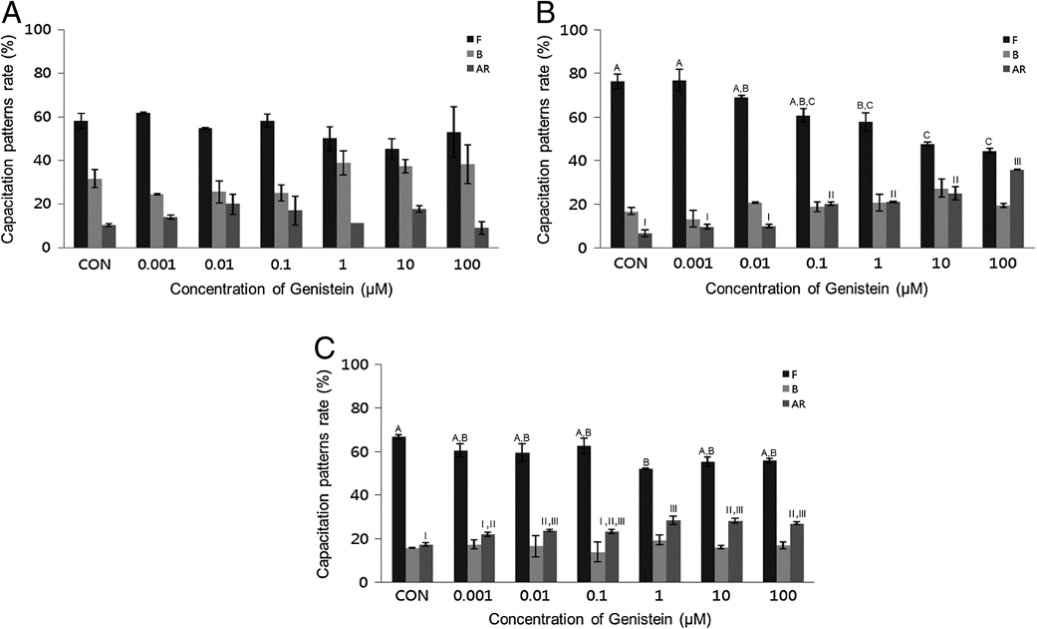
**Effects of 30 min of incubation with genistein (GEN) on capacitation status. (A)** Change of sperm capacitation status of bovine spermatozoa in the absence or presence of GEN (0.001 to 100 μM). **(B)** Change of sperm capacitation status of mouse spermatozoa in the absence or presence of GEN (0.001 to 100 μM). **(C)** Change of sperm capacitation status of porcine spermatozoa in the absence or presence of GEN (0.001 to 100 μM). Capacitation status was distinguished F, B and AR pattern (Black Bar: F pattern, Grey Bar: B pattern, Dark-grey Bar: AR pattern). Data represent mean ± SEM, n =3. ^A, B, C^ Values with different superscripts were significantly different compared to control and the F pattern group, by ANOVA (*P* <0.05). ^I, II, III^ Values with different superscripts were significantly different compared to control and the AR pattern group, by ANOVA (*P* <0.05).

GEN (0.001-100 μM) significantly increased the acrosome reaction in porcine spermatozoa treated for 15 min (*P* <0.05) (Figure 5C), while no detectable effects were observed in mouse spermatozoa (Figure 5B). GEN also significantly increased the acrosome reaction in both mouse and porcine spermatozoa treated for 30 min (*P* <0.05) (Figure 6B and C). A concentration-dependent effect on the acrosome reaction was observed in both mouse and porcine spermatozoa. Capacitation was increased in bovine spermatozoa after 15 min incubation at a concentration of 0.001 μM. Upon treatment with 0.1 μM, this effect gradually decreased with increasing doses of GEN (Figure 5A). No detectable effect was observed after 30 min incubation.

### Effects of OP on bovine, mouse, and porcine spermatozoa

**Figure 7:**
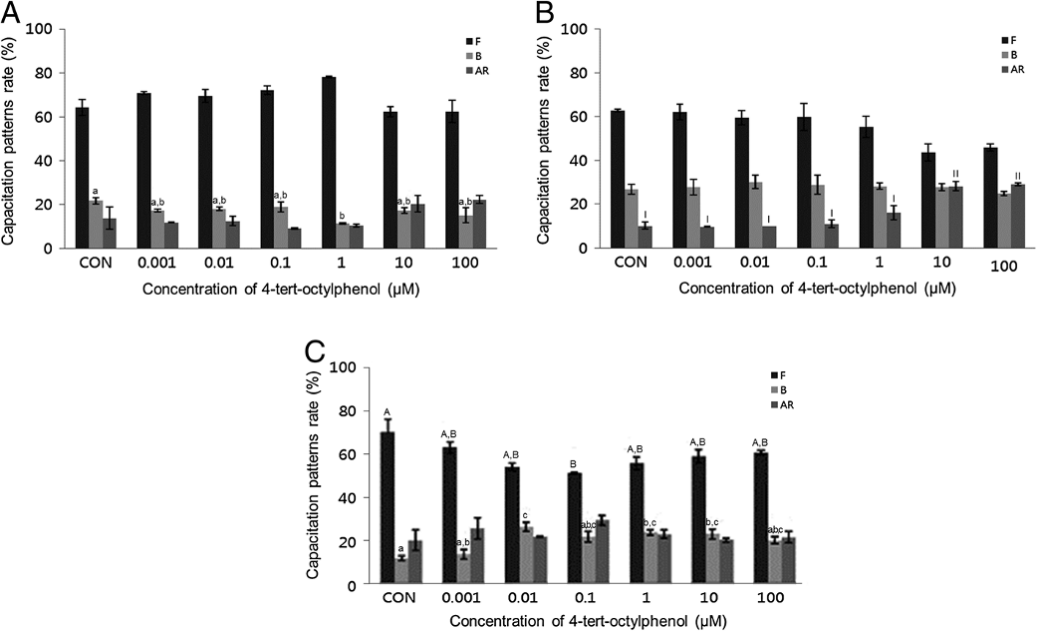
**Effects of 15 min of incubation with 4-tert-octylphenol (OP) on capacitation status. (A)** Change of sperm capacitation status of bovine spermatozoa in the absence or presence of OP (0.001 to 100 μM). **(B)** Change of sperm capacitation status of mouse spermatozoa in the absence or presence of OP (0.001 to 100 μM). **(C)** Change of sperm capacitation status of porcine spermatozoa in the absence or presence of OP (0.001 to 100 μM). Capacitation status was distinguished F, B and AR pattern (Black Bar: F pattern, Grey Bar: B pattern, Dark-grey Bar: AR pattern). Data represent mean ± SEM, n =3. ^A, B^ Values with different superscripts were significantly different compared to control and the F pattern group, by ANOVA (*P* <0.05). ^a, b, c^ Values with different superscripts were significantly different compared to control and the B pattern group, by ANOVA (*P* <0.05). ^I, II^ Values with different superscripts were significantly different compared to control and the AR pattern group, by ANOVA (*P* <0.05).

**Figure 8:**
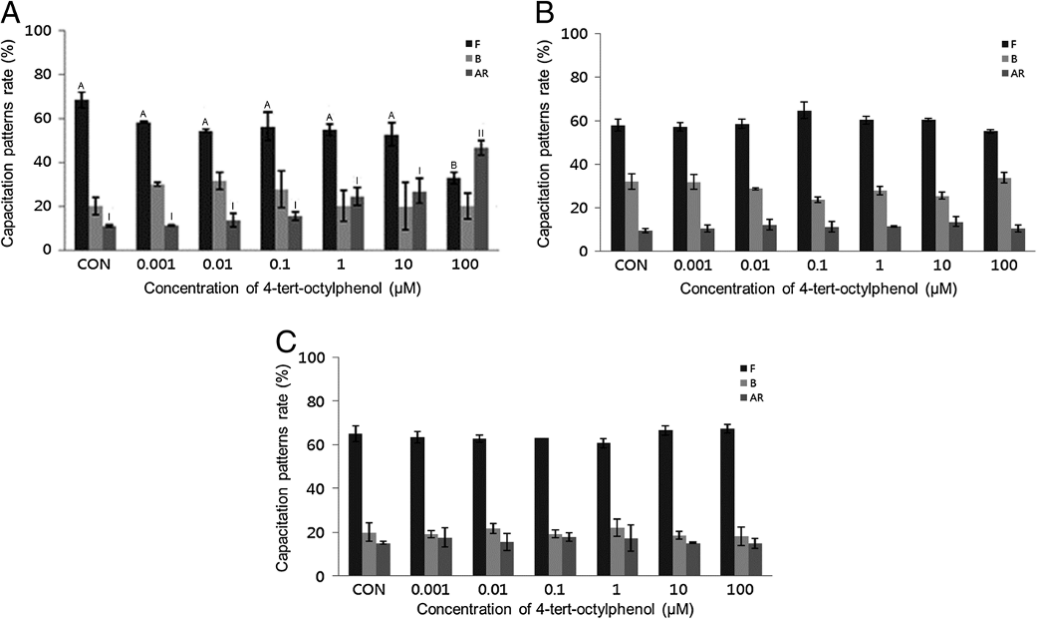
**Effects of 15 min of incubation with 4-tert-octylphenol (OP) on capacitation status. (A)** Change of sperm capacitation status of bovine spermatozoa in the absence or presence of OP (0.001 to 100 μM). **(B)** Change of sperm capacitation status of mouse spermatozoa in the absence or presence of OP (0.001 to 100 μM). **(C)** Change of sperm capacitation status of porcine spermatozoa in the absence or presence of OP (0.001 to 100 μM). Capacitation status was distinguished F, B and AR pattern (Black Bar: F pattern, Grey Bar: B pattern, Dark-grey Bar: AR pattern). Data represent mean ± SEM, n =3. ^A, B^ Values with different superscripts were significantly different compared to control and the F pattern group, by ANOVA (*P* <0.05). ^I, II^ Values with different superscripts were significantly different compared to control and the AR pattern group, by ANOVA (*P* <0.05).

OP (0.001-100 μM) significantly increased the acrosome reaction in mouse spermatozoa after 15 min (*P* <0.05) (Figure 7B). This effect was dose-dependent manner. OP treatment also increased capacitation in porcine spermatozoa incubated for 15 min (Figure 7C). OP increased the acrosome reaction in bovine spermatozoa treated for 30 min, however these differences were not significant except at 100 μM. No detectable effects were observed in mouse, porcine, or bovine spermatozoa (Figure 8).

## Discussion

The present study addressed the question whether estrogens and endocrine disruptors interfere with bovine, mouse, and porcine spermatozoa function. Four chemicals, namely, E2, P4, GEN, and OP were evaluated at concentrations from 0.001 to 100 μM. Uncapacitated spermatozoa were treated for either 15 or 30 min. We then assessed capacitation and the acrosome reaction using CTC analysis.

Interestingly, spermatozoa from mice, porcine, and bovine were responded to E2 in a different way. Mouse and porcine spermatozoa responded at different concentrations and times. Our results from mouse spermatozoa are in accordance with [10]. Unfortunately, we could not compare our findings from bovine and porcine with data from others because, to date, no such data has been collected by others. In various somatic cell systems, E2 has been reported to modulate Ca^2+^ fluxes, generate cyclic nucleotides, activate various kinases and modulate ion channels [14]. cAMP plays a role in spermatozoa physiology, many treatments that accelerate capacitation cause an increase in cAMP. cAMP plays a role in spermatozoa physiology many treatments that acceleration of capacitation cause by increasing in cAMP. Moreover, continuous stimulation of cAMP production appears to be associated with acrosome loss [10], these observations demonstrate the role of E2 in some of the physiological changes that take place in spermatozoa. Estrogens are classically thought to act by binding to estrogen receptors, ESR1 and ESR2 [15]. Notably, several studies have reported the presence of ERs on human [16, 17] and rat [18] cell membranes. GEN and other estrogenic components are able to bind to both estrogen receptors, ESR1 and ESR2 [19–21]. These chemicals may exert their effects by binding to the same receptors. P4 significantly increased the acrosome reaction in both bovine and mouse spermatozoa treated for 15 min (*P* <0.05) (Figure 3A and B), while significantly increased capacitation was observed in porcine spermatozoa (*P* <0.05) (Figure 3C). P4 significantly increased capacitation with dose-dependent manner in mouse spermatozoa treated for 30 min (P <0.05) (Figure 4B), while no detectable effects were observed in either bovine or porcine spermatozoa. Spermatozoa from mice, porcine, and bovine were responded to P4 in the same way as they responded to E2. P4 seems to be involved in the physiological induction of the AR [22]. The changes induced by P4 require intercellular mechanisms dependent on protein kinase C and the Ca^2+^ channel. Unlike Ca^2+^ ionophores, P4 is a physiological inducer of the AR, because it does not bypass normal regulatory mechanisms [23]. In most species, P4 concentration within the reproductive tract fluids is still unknown [24, 25]. In canine spermatozoa, the exposure of P4 binds with its receptors finally correlated with the maturation state of spermatozoa [26]. In stallions, the proportion of spermatozoa with exposed P4 receptors seems to correlate with the fertility of a given animal [27]. However, in bulls, P4 is not involved in the capacitation process, but rather initiates the AR, when spermatozoa are under capacitation conditions [28]. Others have reported that P4 significantly enhances sperm capacitation but does not significantly increase the AR of heparin-capacitated spermatozoa [29]. Therefore, our results indicate that P4 can induce the acrosome reaction in bovine and mouse spermatozoa, as well as stimulating capacitation in mouse and porcine spermatozoa. These findings are in contrast to other reports on bull spermatozoa that said P4 has a potential role in capacitation but not in AR. However, a significant time dependent increases of AR was reported in boar spermatozoa induced by P4 [30]. P4 also failed to accelerate capacitation or acrosome loss in human sperm suspensions pre-incubated for 1 h prior to a 30-min P4 treatment [10]. GEN significantly increased the acrosome reaction in both mouse and porcine spermatozoa treated for 30 min (*P* <0.05) (Figure 6B and C). A concentration-dependent effect on the acrosome reaction was observed in both mouse and porcine spermatozoa. GEN accelerated capacitation but this effect was not significant. Porcine spermatozoa responded to GEN in the same way as mouse spermatozoa did. In porcine spermatozoa, the acrosome reaction was increased at a concentration of 0.01 μM, but mouse spermatozoa required at least 0.1 μM of GEN to elicit a significantly difference. Our results from mouse and porcine spermatozoa are in accordance with those of Fraser et al. [9], who reported that GEN accelerated capacitation and increased the acrosome reaction in human and mouse spermatozoa. While other investigators [19–21] have reported that GEN is able to bind to both estrogen receptors (ERs)-alpha and ER-beta, it has lower affinity than E2. Therefore, these observations were not in accordance with our data, which revealed similar effects for GEN and E2. However, other studies have reported that E2 and GEN both stimulate uncapacitated spermatozoa and that capacitation is associated with an increase in protein phosphorylation in humans [31, 32]. Daidzein, like GEN, is an isoflavone associated with soy isoflavones and has been proven to have the same effect as GEN on uncapacitated mouse spermatozoa. Thus, soy isoflavones-containing products have at least two components with the potential to alter sperm function [33].

Finally, OP significantly increased the acrosome reaction in mouse spermatozoa, in a dose-dependent manner (*P* <0.05) (Figures 7B and 8B), and significantly increased capacitation in porcine spermatozoa treated for 15 min (*P* <0.05) (Figure 7C). Bovine spermatozoa showed significant increases in the AR following 30-minute incubation at the highest concentration of OP studied (*P* <0.05) (Figure 8A). However, no detectable effects were observed in mouse or porcine spermatozoa following incubation for 30 min. While OP has been reported to be estrogenic in fish and mammalian cells and mimics the effect of E2 by binding to the estrogen receptor [34], our data revealed that OP had low estrogenic activity in all spermatozoa tested. This was in accordance with Aydogan et al. [34], who reported that the estrogenic activity of OP was approximately 10^−3^ to 10^−7^ M relative to E2. The low efficiency of OP may result from the lower affinity with which it binds to ERs [19–21]. Luconi et al. [2] also reported that octyphenol polyethoxylate had no detectable effect in humans.

## Conclusions

All chemicals studied effectively altered capacitation and the acrosome reaction in bovine, mouse, and porcine spermatozoa. However, when spermatozoa were incubated for 15 or 30 min in all chemicals studied, capacitation status and acrosome reaction were significantly different in the responsiveness of bovine, mouse and porcine spermatozoa to E2, P4, GEN, and OP were significantly different. Porcine spermatozoa were more responsive than the other spermatozoa. Therefore, we suggest that porcine spermatozoa can be used as a suitable tool for *in vitro* screening of potential endocrine disruptors.
